# Underexpression of *hsa-miR-449* family and their promoter hypermethylation in infertile men: A case-control study

**DOI:** 10.18502/ijrm.v19i1.8177

**Published:** 2021-01-25

**Authors:** Reza Najafipour, Abdolmabood Momeni, Farideh Yousefipour, Shaghayegh Mousavi, Sahar Moghbelinejad

**Affiliations:** ^1^Research Institute for Prevention of Non-Communicable Diseases, Cellular and Molecular Research Centre, Qazvin University of Medical Sciences, Qazvin, Iran.; ^2^Biology Department, School of Basic Science, Arak University, Arak, Iran.; ^3^National Institute of Engineering and Biotechnology, Tehran, Iran.; ^4^Department of Molecular Medicine, School of Medicine, Qazvin University of Medical Sciences, Qazvin, Iran.

**Keywords:** Spermatogenesis, miR-449, Expression, Epigenetic.

## Abstract

**Background:**

Post-transcriptional microRNAs (miRNAs) have a impotrant pattern in the spermatogenesis process.

**Objective:**

Study of the expression and methylation of *hsa-miR-449* family in sperm samples of infertile men.

**Materials and Methods:**

In this case-control study, we recruited 74 infertile men (with asthenozoospermia, teratozoospermia, asthenoteratozoospermia, and oligoasthenoteratozoospermia) and 30 control samles. Methylation-specific PCR (MSP) method was used for methylation evaluation of *hsa-miR-449* *a, b, c *promoter. By Real time PCR (qRT-PCR) method,we showed downregulation of *hsa-miR-449* *a, b, c* in the sperm samples of infertile men and compared it to their fertile counterparts.

**Results:**

There was significant underexperssion, in *hsa-miR-449-b* in oligoasthenoteratospermic samples (p = 0.0001, F = 2.9). About the methylation pattern, infertile men showed high frequency of methylation in the promoter of *hsa-miR-449* *a, b, c* in comparison to controls (60.8% vs 23.3%), the highest amount of methylation was observed in oligoasthenoteratospermia samples (81.2%).

**Conclusion:**

In this study, low expression and high methylation of *hsa-miR-449-b* were observed in infertile men in compared to control samples, which can be one of the causes of defective spermatogenesis.

## 1. Introduction

On average, it is estimated that infertility occurs in 10 to 15% of couples and 50% of infertility cases are due to male factor. The causes of 65-70% of male infertility cases is unknown and the correct mechanism has not been defined (1, 2). Genetic factors can be one of the causes of male infertility (3). But the role of miRNAs in the process of spermatogenesis and male infertility is very important. MiRNAs are non-coding RNAs, and play an important role in regulating gene expression (4, 5). MiRNAs regulate gene expression in two ways: by suppressing transcription and translation (RNAi) (6) or by activating transcription (RNAa) (7-9). The expression of miRNAs was shown in some types of male germ cells (10-14). The *hsa-miR-449* was first detected in the fetal brain of mice(15, 16).

The *hsa-miR-449* family has three members in mice and human, namely *hsa-miR-449a*, *hsa-miR-449b*, and *hsa-miR-449c*. These miRNAs have conserved sequence among different species and are located on the second intron of the *Cdc20b* gene. While the three *hsa-miR-499* (*miR-499 a, b, c*) members have the same seed sequences, *hsa-miR-449* members play the main role in the control of cell cycle and differentiation of epidermis (17-19).

In this regard, studies have shown that the *hsa-miR449* family (*hsa-miR-449a*, *hsa-miR449b*, and *hsa-miR-449c*) and the *hsa-miR-34 b, c* (*hsa-miR-34b-3p* and *hsa-miR-34c-5p*) contain an identical seed sequence and have same sequence with the another miRNA, *hsa-miR-34a-5p* (17-21). About in addition, with respect to the role of hsa-miR-449 in spermatogenesis and male infertility, it was shown that the *hsa-miR-449* family highly express in spermatocyte, spermatid, and adult testis. In one study was reported that, inactivation of both the *hsa-miR34-b,c* and *hsa-miR-449* causes low sperm counts, motility and high abnormal sperm morphology in animal models (22). CpG methylation of mentioned genes is one possible reason for the their underexpression (20, 22), so that, under expression and high methylation of *hsa-miR-449* (*a, b, c*) (as an important tumor-suppressor gene) have been shown in various cancers (23, 24). Besides, methylation of their promoter region has also been shown to be one of the mechanisms of expression reduction in adition to playing an important role in carcinogenesis of these microRNAs. Up to now, the patterns of *hsa-miR-449* family expression and methylation has not been reported in different groups of infertile men and all the information in this regard has been based on animal models (25). Therefore, in this study we evaluated the expression and methylation pattern of *hsa-miR-449* family in infertile men.

## 2. Materials and Methods

### Subject recruitment and sampling 

In this case-control study, 74 infertile men with idiopathic asthenozoospermia (n = 14), teratozoospermia (n = 16), asthenoteratozoospermia (n = 28), and oligoasthenoteratozoospermia (n = 16) were collected during 2018-2019 from the ACECR Telemedicine Infertility Center Qazvin, IRAN, based on the WHO criteria. The condition of infertile men was as follows: a history of infertility for at least 1 yr with their wives having a normal gynecological evaluation. However, infertile men conditions such as cystic fibrosis, Klinefelter syndrome, varicocele, chemotherapy, AZF, and genes micro deletions were not included in this study. In addition, 30 fertile healthy men were recruited as the control group.A questionnaire was designed to evaluation of the patients and controls' information, including medical history, occupational and environmental condition, smoking condition (an adult who has smoked 100 cigarettes in his lifetime and who currently smokes cigarettes)and reproduction status (Table I). Patients were advised not to have sexual abstinence for three days before sampling. After sampling, semen samples were stored at 37°C for 30 min to complete the liquefaction. Then, based on WHO criteria sperms concentration, motility, and morphology were evaluated (26).

### RNA extraction and qRT-PCR

After liqufication, we centrifuged the semen samples for 10 min at 500 g. Then 1 ml FSB (Merck, Germany) was added to sperms pellet and somatic cells removed.TRIZOL reagent was used for isolation of the total RNA from the sperms based on kit protocols (Invitrogen Life Technology Co., USA). *hsa-miR-449-a* (MI0001648), *hsa-miR-449-b* (MI0003673), and *hsa-449-c* (MI0003823) were studied in this research. *hsa-miR30a-5p* (MIMAT0000087) and *hsa-miR100-5p* (MIMAT 0000102) were used as internal controls. Rotor gene-Q real-time PCR system (Qiagene, Germany) was used to quantifing of the RNA expression. Total amount of master mix was 10 µl and included 1 µl of reverse and forward primers (Exiqon, Denmark), 5 µl of Ampliqon real Q plus 2× master mix green (Ampliqone, Denmark), and 4 µl of diluted cDNA. For enzyme activation we incubated master mix for 15 min at 95°C. Then reaction was runned in 40 cycle for 20 sec at 95°C and 60 sec at 60°C. Ct values was used for evaluation of expression rate of studied miRNAs. *hsa-miR30a-5p* and *hsa-miR100-5p* were used as the endogenous controls. The 2${}^{-▵\mathrm{Ct}}$ method used for expression rate detection of target genes in comparision to internal controls.

### DNA extraction and bisulfite modification 

Phenol-chloroform method was used for DNA extraction. 2-5 µg of extracted DNA was bisulfited by using of EpiJET${}^{\mathrm{TM}}$ Bisulfite Conversion kit (Thermo Fisher Scientific, Inc).

### MSPCR 

The *hsa-miR-449 methylation *status was evaluated in all studied samples. For the targeted site, methylation-specific primers were designed. While the methylated primers were Forward: 5'-CGTTCGTTAATTTTTTCGTTTTTTGTCGC-3') and Reverse: 5'-GTCAAAACCCGAATAAAATTCCCCG ACG-3', the unmethylated primers were Forward: 5'-TTGTTTGTTAATTTTTTTGTTTTTTGTTGT-3' and Reverse: 5'-ATCAAAACCCAAATAAAA TTCCCCAACA-3'. Methylation-specific PCR (MSP) was used to evaluation of methylation status of *hsa-miR-449-abc promoter region.* 1 µL of bisulfit converted DNA with methylated and unmethylated primers was amplifiedin in final 10 µL reaction mixture. “The PCR conditions for methylation status were: 95°C for 15 min (Hot start), followed by 35 cycles at 95°C for 20 sec (denaturation), 56.5°C for 45 sec (annealing), and 72°C for 45 sec (extension). PCR condition for unmethylated-primers was the same as the methylated condition except for the number of cycles and annealing temperature (60°C)" (27).

**Table 1:** Characterization of studied samples


**Parameters**		**Fertile**	**Asthenospermia**	**Teratospermia**	**Astheno-teratospermia**	**Oligo-astheno-teratospermia**
**Age (yr) **		31.2 ± 1.8	36.1 ± 2.2	32.2 ± 5.5	34.1 ± 4.4	38.1 ± 4.1
**Liquefaction time (min) **		24 ± 12.2	22.2 ± 6.6	25 ± 8.9	25 ± 10.3	23.7 ± 8.06
**Testicular volume (ml)**		22 ± 3.2	21.2 ± 1.8	20.1 ± 1.8	19 ± 2.1	19.8 ± 3.1
**Ejaculate volume (ml)**		3.2 ± 1.5	2.8 ± 1.9	2.3 ± 1.8	2.9 ± 1.2	3.1 ± 2.2
**Viscosity (%)**				
	**Normal **	86.7	88.9	75	78.6	81.3
	**Somewhat**	10	11.1	25	17.9	18.8
	**Thick**	0	0	0	3.6	0
	**Very thick**	3.3	0	0	0	0
**Concentration (106/ml)**		244.4 ± 13.08	178.8 ± 54.9	219.2 ± 108.3	112.86 ± 87.7	13 ± 6.8
**Progressive motility (A+B) (%)**		34.5 ± 8.05	13.8 ± 4.8	42.8 ± 2.5	8.14 ± 5.9	4.19 ± 5.07
**Normal morphology (%)**		11.8 ± 4.12	5.1 ± 1.5	2.7 ± 0.44	1.7 ± 0.78	1.2 ± 0.68
* Mean ± SD						

### Ethical considerations

This case-control study was approved by the Ethics Committee of Qazvin university of medical science with dedicated ID IR.QUMS.REC.1396.294. The recruited patients gave their informed written consent.

### Statistical analysis

The GraphPad software (GraphPad PRISM V 5.04) was used for the data analysis. The analysis of variance test (ANOVA) was used for the evaluation of the miRNAs expression levels difference among the different studied groups. The frequency of promoter methylation pattern was evaluated by a nonparametric test (Kruskal-Wallis). The correlation between the miRNA expression rate, methylation with different sperm parameters was analyzed by Spearman's rank correlation. All P-values were two-tailed, with p < 0.05 considered as statistically significant.

## 3. Results

### Expression of miRNAs in sperm samples of studied groups

**Figure 1:**
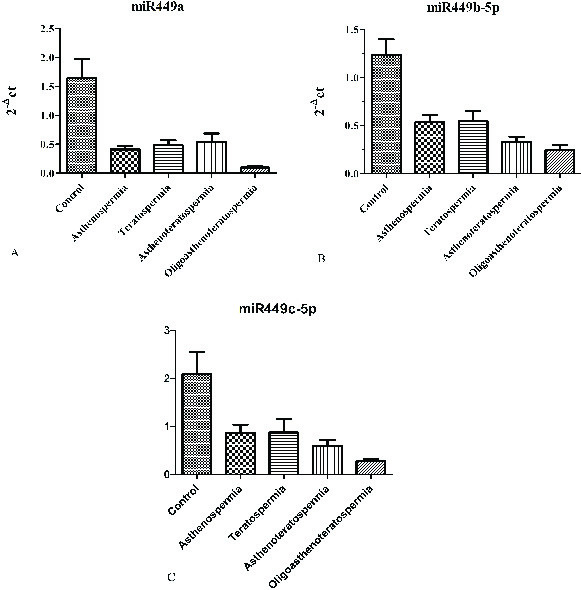
The expression ratio (2-ΔΔ ct ) of *hsa-miR-449-a, b, c* in the sperm samples of fertile controls and idiopathic infertile males. (A) The expression ratio of *has-miR-449-a* in asthenospermia, teratospermia, asthenotheratospermia and oligoasthenoteratospermia compared to the control fertile men. (B) The expression pattern of *hsa-miR-449-b* in asthenospermia, teratospermia, asthenotheratospermia, oligoasthenoteratospermia compared to the control fertile men. (C) The expression ratio of *hsa-miR-449-c* in asthenospermia, teratospermia, asthenotheratospermia, oligoasthenoteratospermia compared to the control fertile men. Data are presented as Tukey's box plots showing the median and mean (+) values.

**Figure 2:**
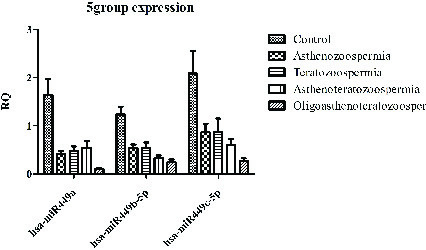
The expression pattern of miR-449-a,b,c in all studied groups: Fertile (n = 30) and infertile (n = 74) groups. Values are presented as Mean ± SD.

About the expression rate, we saw significant downregulation of the *hsa-miR-449a* expression in infertile group (0.24 ± 0.2) in comparison to the control groups (0.98 ± 0.37) (p = 0.0001). Also, significant down-egulation of this miRNA was shown in oligoasthenoterathospermia (0.05 ± 0.02), asthenospermia (0.24 ± 0.11), terathospermia (0.28 ± 0.2), and asthenoterathospermia (0.3 ± 0.11) as compared to the control group (0.98 ± 0.37) (F = 7.1 p = 0.0001). MirRNA expression ratio had not significant difference among the four infertile groups, (p = 0.21) (Figure 1A). The expression of *hsa-miR-449b* was downregulated significantly among the oligoasthenoterathospermia groups (0.022 ± 0.01) in comparison to the asthenospermia (0.04 ± 0.02), terathospermia (0.04 ± 0.032), asthenoterasthospermia (0.03 ± 0.02), and the control group (0.12 ± 0.09) (p = 0.0001, F = 2.9) (Figure 1B). However, in the case of *hsa-miR-449c*, oligoasthenoterathospermia patients showed significant downregulation of this miRNA (0.04 ± 0.03), also we observed this downregulation among the asthenospermia (0.15 ± 0.14), terathospermia (0.15 ± 0.14), and asthenoterasthospermia (0.1 ± 0.09) groups in compared to the control group (0.37 ± 0.01) (F = 5.04, p = 0.001) (Figure 1C). The average of the expression of the three studied miRNAs in fertile men showed that *hsa-miR-449a* had the highest levels of expression in these individuals (0.98 ± 0.3), followed by *hsa-miR-449c* (0.37 ± 0.01) and *hsa-miR-449b* (0.12 ± 0.09) (Figure 2). As displayed in Figure 2, among the three studied miRNAs, *hsa-miR-449b* had the lowest expression ratio in infertile men especially in oligoasthenotaratospermic men (0.022 ± 0.01).

### Investigating the relationship between miRNAs expressions and parameters of sperm 

**Figure 3:**
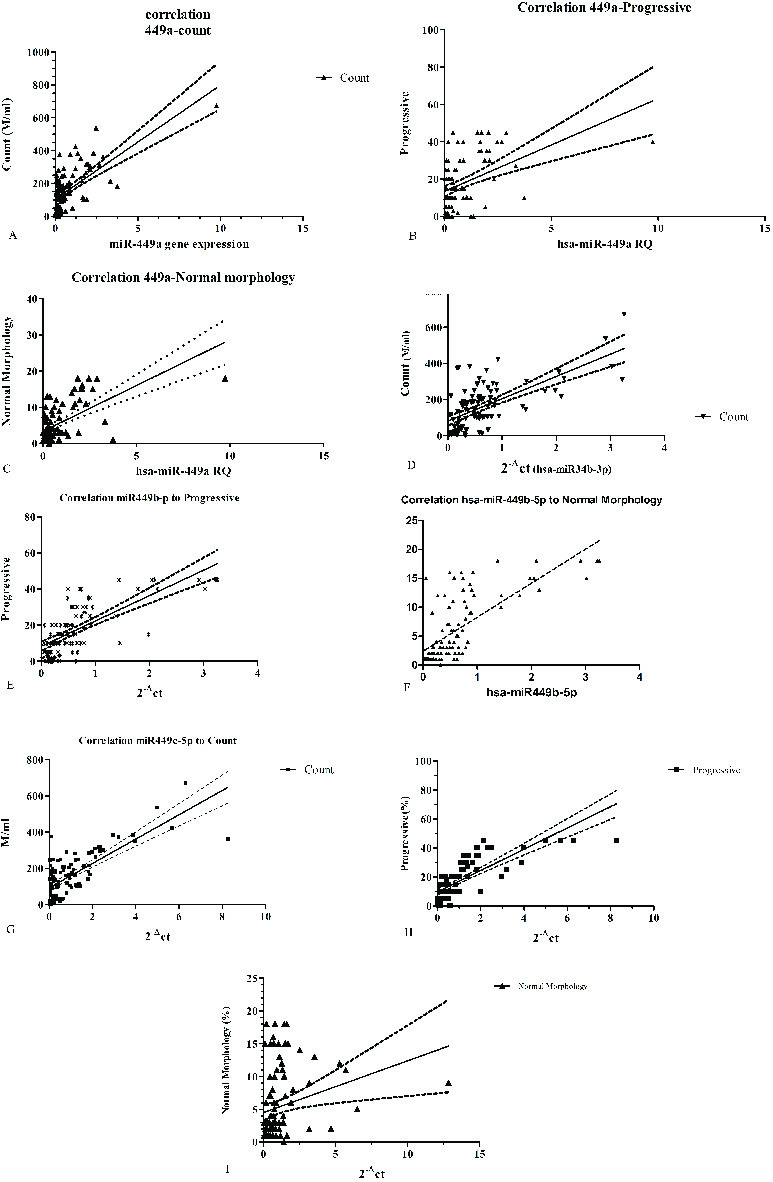
Correlation between the *hsa-miR-449-a* expression ratio with (A) count (r = 0.2, p= 0.039), (B) progressive motility (r = 0.44, p = 0.000), and (C) normal morphology (r = 0.45, p = 0.0001). Correlation between the *hsa-miR-449-b* expression ratio with (D) count (r = 0.386, p = 0.0001), (E) progressive motility (r = 0.556, p = 0.0001), and (F) normal morphology (r = 0.55, p = 0.0001). Correlation between the *hsa-miR-449-c* expression ratio with (G) count (r = 0. 662, p = 0.012), (H) progressive motility (count = 0.52, p = 0.001), and (I) normal morphology (count r = 0. 63, p = 0.0001).

In all studied samples, we observed significant correlation between the expression of *hsa-miR-449a* and the sperm progressive motility (r = 0.44, p = 0.0001), sperm count (r = 0.2, p = 0.03), and normal morphology (r = 0.46, p = 0.0001). The pearson test results showed, significant correlation between the expression of *hsa-miR-449b* miRNA and the spem progressive motility (r = 0.55, p = 0.0001), sperm count (r = 0.59, p = 0.0001), and normal morphology (r = 0.58, p = 0.0001). Also, there was significant correlation between the sperm count (r = 0.38, p = 0.0001), progressive motility (r = 0.22, p = 0.0322), and normal morphology (r = 0.32, p = 0.0001) with *hsa-miR-449c experssion* among all studied samples (Figure 3 A-I).

### The methylation patterns of the promoter region of the *hsa-miR-449a,b,c*

**Figure 4:**
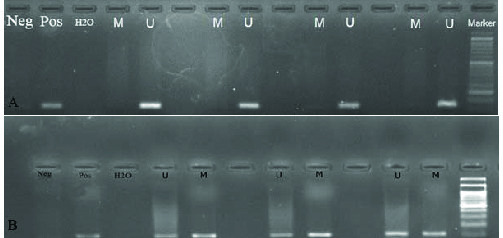
DNA methylation status of the promoter of *hsa-miR-34bc* sperm samples of fertile and infertile patients. (A) Sperm DNA from fertile control samples. In most samples only the unmethylated allele was amplified, however, as can be seen in the figure, some samples showed both methylated and unmethylated alleles. (B) In this image, samples of patients with asthenoteratospermia, oligoasteniteratospermia is presented, in all samples there are unmethylated/methylated bands. As seen, the severity of methylated bands is very high.

MSP results showed that, 60.8% and of the patients and 23.3% of the controls had methylated allele (p = 0.0001). The unmethylated allele was detected in all patients and controls. Oligoasthenoteratospermic (81.2%) and asthenothratospermic (61.2%) patients showed the highest frequency of methylation (Table II) (Figure 4 A, B). There was not significant correlation between *hsa-hsa-miR-449abc* promoter methylation with the liquefaction time (r = 0.022, p = 0.065) and viscosity (r = 0.969, p = 0.65). However, we observed a significant negative correlation between sperm count (r = -0.235, p = 0.003), progressive motility (r = -0.375, p = 0.0001), and normal morphology of sperms (r = -0.356, p = 0.0001) with methylation pattern of *hsa-miR-449abc* promoter. Also, there was significant negative correlation between methylation of *hsa-miR-449abc* promoter region and *hsa-miR-449a* (r = -0.110, p = 0.02), *hsa-miR-449b *(r = -0.245, p = 0.01), and *hsa-miR-449c* (r = -0.348, p = 0.005) expression ratio (Table III). *The results of the effect of smoking on methylation status of mir-449-abc promoter showed* the high frequency of methylation in men who smoked (87.7%) in comparison to men who did not (75.2%, χ${}^{2}$ = 4.2, p = 0.003).

**Table 2:** Frequencies of miR-449-a,b,c methylation statues in different studied groups


	**Unmethylated (%)**	**Methylated/unmethylated (%)**	**P-value**
**Fertile**	76.7	23.3	
**Infertile**	39.2	60.8	0.0001
**Asthenospermia**	29.8	70.2	0.0001
**Teratospermia**	41.8	58.2	0.0001
**Asthenoteratospermia**	38.8	61.2	0.0001
**Oligoasthenotheratospermia**	18.8	81.2	0.0001
P-value indicate significant difference in the frequency of methylated allele between each group of infertile men in compared to the fertile ones. Nonparametric test (Kruskal-Wallis)			

**Table 3:** Correlation between miR-449-a,b,c methylation statues with different semen parameters and miR-44-a, miR-449-b, miR-449-c expression rate


	**Liquefaction**	**Viscosity**	**Count**	**Motility (A+B)**	**Normal Morph**	**miR-449-a 2${}^{-}{}^{▴\mathrm{ct}}$**	**miR-449-b 2${}^{-}{}^{▴\mathrm{ct}}$**	**miR-449-c 2${}^{-}{}^{▴\mathrm{ct}}$**
**miR-449-a,b,c**	0.011 (0.0863)	1. 03 (0.15)	-0.348 (0.000)	-0.378 (0.000)	-0.356 (0.000)	-0.51 (0.011)	-0.428 (0.003)	-0.276 (0.001)
*Spearman${}^{{}^{'}}$s rank correlation test								

## 4. Discussion

In our project, significant downregulation of the *hsa-miR-449abc* was seen in the sperm samples of infertile men. Briefly, miRNAs have the main role in incidence of male infertility through the gene expersion regulation (22). The miR-449 family have three members in mice and humans, *hsa-miR-449a*, *hsa-miR-449b*, and *hsa-miR-449c*. Three *hsa-miR-499 (miR-499a, b, c)* members are transcribed simultaneously and have the same seed sequence (17, 20).

The roles of *hsa-miR-449* family in human reproduction, so far it has only been based on the results of animal models studeis (21). These studies report that *hsa-miR-449* family is one of the most upregulated testicular miRNA and have major role in the initiation of meiotic phase in the adult testes. Also, this group showed that “hsa-miR-449 is predominantly and exclusively expressed in spermatocytes and spermatids in the adult testes” (21). Liu and colleague showed a presence of *hsa-miR-449* family in spermatozoa, but their absence in oocytes (28). In the line of above study, our results showed expression of these miRNAs was shown in sperm${}^{,}$s s of the control (fertile) group, and *hsa-miR-449a* had the highest levels of expression. We also saw a significant downregulation of these miRNAs in the sperm samples of infertile patients, and *hsa-miR-449b* had the highest reduction. Another study reported abnormal different sperm parameters in *hsa-miR-34bc/449* knock-out mice with oligoteratoasthenospermia. They reported that deficient mice had spermatozoa maturation stages problme, but *hsa-miR-449* or *hsa-miR-34* cluster Knockout (KO) mice showed no explained phenotype (22). This is because *hsa-miR-449* and *hsa-miR-34b/c* have the same “seed sequence,” which maps between the second and seventh nucleotides; this core element is necessary for base pairing with target mRNAs, and can target the same set of mRNAs. On the other hand, *hsa-miR-449* and *hsa-miR-34b/c* have the same expression profiles within testicular formation, and they are located to the precise identical spermatogenic cell types, such as spermatocytes and spermatids. In this regard, it is better in future studies that the expression pattern of *hsa-miR-449* family and *hsa-miR-34b/c* be evaluated simultaneously. Interestingly, along with Comazzetto and colleague's 2014 study, the highest downregulation of *hsa-miR-449* family in oligoteratoasthenospermic men was observed, also we saw significant correlation between expression of this gene family and sperm progressive motility, count, and normal morphology (29).

Methylation is one of the mechanisms in expression regulation of miRNAs (30). Expression and methylation of the *hsa-miR-449a,b,c *as the tumor suppressor genes has been reported in different cancers such as prostate (31), hepatocellular carcinoma (32), and osteosarcoma (18). Also, the role of methylation in regulating the expression of miRNAs involved in spermatogenesis and infertility has also been demonstrated (33). To the best of our knowledge, there is no clinical data on the methylation status of *hsa-miR-449a, b, c* promoter in human sperm. In the study of *hsa-miR-449*family methylation, our results showed that, there was high frequency of methylation in infertile men (60.8%) specially in the oligoasthenoteratospermia patients. Interestingly, oligoasthenoteratospermia patients had the highest gene expression reduction. Also, in this research we observed a negative correlation between underexperssion and hypermethylation of *hsa-miR-449a, b, c*. This suggests that hypermethylation can be one of the downregulation mechanisms of these gene family. Further, we observed hypermethylation of promoter region among the 87.7% of smokers. Smoking and obesity have the major effect on the quantity of sperm miRNA (34). Recently, in the sperm samples of men exposed to lifestyle stress Dickson *et al.* reported under expression and hyper methylation of *hsa-miR-449* and *hsa-miR-34* (35). Acording to what was said, it is better to study the effect of other environmental factors on the rate of the *hsa-miR-449a, b, c* methylation.

## 5. Conclusion

Results of this research showed under-experssion and hypermethylation of the *hsa-miR-449* a,b,c in sperm samples of infertile men. According to the results of this study, it can be said that one of the possible causes of defective spermatogenesis in infertile people can be reduced expression and increased methylation of these miRNAs family.

##  Conflict of Interest

The authors declare that there is no conflict of interest.
